# Test-retest reliability and minimal detectable change of the Beck Depression Inventory and the Taiwan Geriatric Depression Scale in patients with Parkinson's disease

**DOI:** 10.1371/journal.pone.0184823

**Published:** 2017-09-25

**Authors:** Sheau-Ling Huang, Ching-Lin Hsieh, Ruey-Meei Wu, Wen-Shian Lu

**Affiliations:** 1 School of Occupational Therapy, College of Medicine, National Taiwan University, Taipei, Taiwan; 2 Division of Occupational Therapy, Department of Physical Medicine and Rehabilitation, National Taiwan University Hospital, Taipei, Taiwan; 3 Department of Neurology, National Taiwan University Hospital, College of Medicine, National Taiwan University, Taipei, Taiwan; 4 School of Occupational Therapy, Chung Shan Medical University, Taichung, Taiwan; 5 Occupational Therapy Room, Chung Shan Medical University Hospital, Taichung, Taiwan; Chinese Academy of Sciences, CHINA

## Abstract

**Background:**

The Beck Depression Inventory II (BDI-II) and the Taiwan Geriatric Depression Scale (TGDS) are self-report scales used for assessing depression in patients with Parkinson’s disease (PD) and geriatric people. The minimal detectable change (MDC) represents the least amount of change that indicates real difference (i.e., beyond random measurement error) for a single subject. Our aim was to investigate the test-retest reliability and MDC of the BDI-II and the TGDS in people with PD.

**Methods:**

Seventy patients were recruited from special clinics for movement disorders at a medical center. The patients’ mean age was 67.7 years, and 63.0% of the patients were male. All patients were assessed with the BDI-II and the TGDS twice, 2 weeks apart. We used the intraclass correlation coefficient (ICC) to determine the reliability between test and retest. We calculated the MDC based on standard error of measurement. The MDC% was calculated (i.e., by dividing the MDC by the possible maximal score of the measure).

**Results:**

The test-retest reliabilities of the BDI-II/TGDS were high (ICC = 0.86/0.89). The MDCs (MDC%s) of the BDI-II and TGDS were 8.7 (13.8%) and 5.4 points (18.0%), respectively. Both measures had acceptable to nearly excellent random measurement errors.

**Conclusions:**

The test-retest reliabilities of the BDI-II and the TGDS are high. The MDCs of both measures are acceptable to nearly excellent in people with PD. These findings imply that the BDI-II and the TGDS are suitable for use in a research context and in clinical settings to detect real change in a single subject.

## Introduction

People with Parkinson’s disease (PD) are clinically associated with movement-related symptoms (e.g., tremor, bradykinesia, rigidity, and postural instability). The recent literature shows that the impact of non-motor symptoms on daily function in the patients with PD is notable [1–3]. Depression is one of the most prevalent neuropsychiatric symptoms in patients with PD [4, 5]. Among non-motor symptoms, depression is one that has a great impact on the quality of life of patients with PD and their caregivers [3, 6, 7]. The assessment of depression can help clinicians detect early stages of depression in patients with PD and plan appropriate intervention. To screen and monitor the state of depression, clinicians need to routinely assess the depression in patients with PD. Moreover, to interpret the results of the assessments, clinicians have to decide whether the change scores in the depression scales are true, or beyond measurement error.

Test-retest reliability refers to the extent of agreement between measurements of a measure in successive sessions [8]. In addition, random measurement error exists in all assessments. If the measurement error of a measure is not determined, the interpretability of the test scores is limited [9, 10]. To calculate the random measurement error, the minimal detectable change (MDC) is used. The MDC, also called the smallest real difference (SRD) [11], is the smallest threshold of a change score that is beyond random error at a certain confidence level (usually 95%) [12]. The MDC can be used as a threshold to confirm whether a difference between consecutive assessments on a measure of a single patient indicates a real change or is due to random error [13]. Thus, the value of MDC also indicates the amount of random measurement error. Accordingly, the MDC of a measure is vital in the interpretation of data for both clinicians and researchers [9, 10].

The second edition of the Beck Depression Inventory (BDI-II) is widely used for screening, assessing, and monitoring the depression status and change trend in community-dwelling older adults [14] and in different populations [15–17]. The Taiwan Geriatric Depression Scale (TGDS) is frequently used to screen for depression in the elderly [18] and to assess depression in the elderly with depressive disorder [19]. To our knowledge, the test-retest reliability and the MDC of the TGDS have not been examined in people with PD. Although the test-retest reliability and the MDC of the BDI-II in people with PD have been investigated, the characteristics of the participants in the data analysis of test-retest reliability and MDC have not been clearly reported, limiting the generalization of these results [20]. A measure without a determined MDC does not allow clinicians and researchers to explain change scores in successive measurements reasonably. That is to say, lacking explicit MDCs, the BDI-II and the TGDS have limited applicability and interpretability in people with PD. Therefore, the aim of this study was to estimate and compare the extents of the test-retest reliability and the amounts of random measurement error of the two measures. Our results should help potential users to select appropriate measures for, and interpret the clinical data of, patients with PD.

## Materials and methods

### Participants

All of the participants in this study were recruited from special clinics for movement disorders in the Department of Neurology of a medical center located in northern Taiwan. The inclusion criteria were as follows: (1) Diagnosis of PD by a movement disorder specialist, based on the UK PDS Brain Bank Criteria for the diagnosis of PD; (2) Hoehn and Yahr stages between stages I and III; (3) ≥ 50 years; (4) Mini-mental state examination (MMSE) score > 20 points; and (5) agreement to participate in this study and signing of the consent forms, as approved by the medical ethics committee at the medical center. Participants were excluded from the study if they had any of the following conditions: (1) Atypical Parkinsonism, including diffuse Lewy body dementia, multiple system atrophy, progressive supranuclear palsy, and corticobasal degeneration; (2) Secondary Parkinsonism (e.g., vascular Parkinsonism); or (3) Dementia, based on the diagnostic criteria for research in the ICD-10 Classification of mental and behavioral disorders. We retrieved medical records of the patients to confirm whether they had been diagnosed with the above diagnoses by their physicians.

### Procedures

The participants were screened and invited by the movement disorder specialists. All participants filled out two questionnaires during the “on” status at the same place in two sessions about 2 weeks apart. Half of the participants filled out the BDI-II before the TGDS; the order was reversed for the other half to control possible bias of the testing sequence. Prior to the second session assessment, the research assistant confirmed that each participant had no change of the medication for Parkinson’s disease and other neuropsychiatric disorders; no change of Parkinson features, such as tremor, rigidity, bradykinesia, and gait disturbance; and no experience of brain injury during the follow-up period of this study.

### Measures

#### Beck Depression Inventory (BDI-II)

The BDI-II has 21 items scored on a 4-point scale (from 0 to 3), and a total score ranging from 0 to 63. In accordance with the BDI-II manual, the severity of depression was distinguished by the total score of the BDI-II as minimal (0–13), mild (14–19), moderate (20–28), or severe (29–63) [21].

#### Taiwan Geriatric Depression Scale (TGDS)

The Taiwan Geriatric Depression Scale (TGDS), based on the Geriatric Depression Scale, is a sensitive, valid, and reliable measure for use in elderly outpatients in Taiwan [22]. It consists of 30 items of 1 point each, with a higher score indicating more severe depression. It has adequate test-retest reliability, and the best cutoff value for depressive status for geriatric outpatients is 15 [22].

### Data analysis

SPSS 16.0 (SPSS Inc., USA) was employed to analyze our data. We first investigated the test-retest reliability of the measures by calculating the intraclass correlation coefficient (ICC). An ICC value of more than 0.80 is considered to indicate high test-retest reliability of a measure [23].

The value of ICC was then employed to estimate the standard error of measurement (SEM), and thereafter, to estimate the MDC (95% level of confidence) [9, 12].

The MDC% was then calculated by dividing the MDC by the maximal score of the measure [24]. An MDC% < 30% is considered acceptable, and < 10% is considered excellent [24].

In addition, we made Bland-Altman plots with 95% limits of agreement (LOA) [25] to observe the variance between repeated assessments. Assuming the differences follow normal distribution, 95% of the differences would distribute between mean difference ± 1.96 SD_difference_ (i.e., LOA). The plots are useful to present heteroscedasticity. Heteroscedasticity means that a trend of increasing or decreasing exists in the amounts of difference within two adjacent assessments as the mean scores of the two assessments increase [26]. We used Pearson’s *r* to examine whether heteroscedasticity existed by calculating the level of association between the average scores and the absolute values of difference of adjacent assessments. If Pearson’s *r* > 0.3, the data were considered heteroscedastic [27].

We used paired t tests to examine whether systematic bias existed between test-retest assessments.

Last, we investigated the correlation between the scores of the BDI-II and the TGDS using the Pearson correlation coefficient (*r*) at first and second section assessments, respectively. The Pearson *r* ≥ 0.8 was considered to show very strong correlation [28].

### Ethics

Ethics approval was obtained from Research Ethics Committee, National Taiwan University Hospital—Project Number: 200705055R.

## Results

Seventy-five participants with PD agreed to participate in the study. Five participants were lost to follow-up due to loss of contact or refusal to re-test. The mean baseline scores of the TGDS between the remaining 70 participants and the 5 participants lost to follow-up were not statistically different (p = 0.116), but the difference of the BDI-II between the 70 participants and the 5 patients lost to follow-up was statistically significant (p = 0.037). Demographic and clinical characteristics of the 70 participants are shown in Table 1. Their mean age was 67.7 years, and 63.0% of the participants were male. The mean baseline scores of the BDI-II and the TGDS were 8.5 points and 8.2 points, respectively.

**Table 1:** The demographic and clinical characteristics of the participants.

Characteristic	Participants completing two assessments (n = 70)	Participants lost to follow-up (n = 5)
Age, mean (SD)	67.7 (8.8)	66.4 (3.8)
Sex, male/female (n)	44/26	4/1
H&Y stage		
I II III (n)	17 29 24	3 1 1
PD evolution time (range)	6 months to 15 years	6 months to 10 years
BDI-II baseline scores (point), mean (SD)	8.5 (8.4)	2.6 (0.9)
TGDS baseline scores (point), mean (SD)	8.2 (6.0)	6.0 (9.0)
Interval between two assessments (day), mean (SD)	13.3 (2.7)	-

BDI-II: the Beck Depression Inventory (II)

TGDS: the Taiwan Geriatric Depression Scale

The test-retest reliability indices of the BDI-II and the TGDS are shown in Table 2. The ICCs for the BDI-II and the TGDS were 0.86 and 0.89, respectively. The mean differences for the BDI-II and the TGDS were 0.5 and -0.4, respectively. The MDCs (MDC%) of the BDI-II and the TGDS were 8.7 points (13.8%) and 5.4 points (18.0%), respectively.

**Table 2:** The test-retest reliability indices of the BDI-II and the TGDS (n = 70).

Test	First session Mean (SD)	Second session Mean (SD)	Difference Mean (SD)	ICC (95% CI)	MDC (MDC %)
BDI-II	8.5 (8.4)	9 (8.5)	0.5 (4.5)	0.86 (0.78–0.91)	8.7 (13.8%)
TGDS	8.2 (6.0)	7.8 (6.5)	-0.4 (2.9)	0.89 (0.83–0.93)	5.4 (18.0%)

BDI-II: the Beck Depression Inventory (II)

TGDS: the Taiwan Geriatric Depression Scale

ICC: intraclass correlation coefficient

**ICC**_**(2,1)**_ **= (BMS–EMS)/(BMS + EMS + 2(JMS–EMS)/n)**, BMS = between-participants mean square, EMS = the error mean square, JMS = observations mean square, and n = the number of participants

MDC: minimal detectable change

$\text{MDC}=1.96\times \text{SEM}\times \sqrt{2};$ MDC% = (MDC/ maximal score of measurements) x 100%

**Fig 1:**
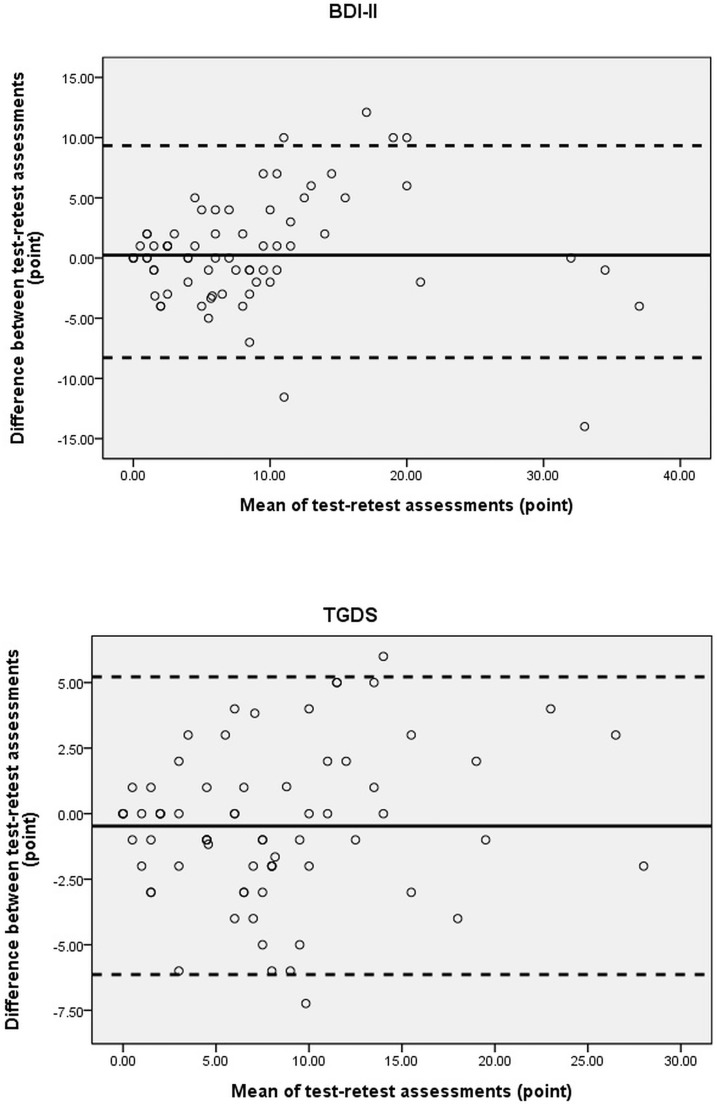
Bland & Altman plots of the difference scores against the mean scores of each pair of the BDI-II and the TGDS. The solid line represents the mean of the differences. The 2 dashed lines define limits of agreement (mean of the difference ± 1.96 SD).

In Fig 1, the differences of scores are plotted against the mean scores of the 2 measurements of both measures [25]. The LOAs (limits of agreements) ranged from 9.3 to -8.3 points for the BDI-II, and from 5.2 to -6.1 points for the TGDS. In addition, the Pearson’s correlation coefficients (r) between the mean and the difference for the BDI-II and the TGDS were 0.4 and 0.3, respectively.

The results of paired t tests showed that the differences between the two sessions of the BDI-II and the TGDS were not significant (p > 0.05).

The Pearson’s *r* values were 0.80 and 0.85 at the first and second sections, between the scores of the BDI-II and the TGDS, respectively. The results indicated that the correlation between the BDI-II and the TGDS was very strong.

## Discussion

We compared the extent of the test-retest reliability of the two measures. The results showed that the ICC values of the BDI-II and the TGDS were high (ICC = 0.86/0.89). Our findings indicated that both the BDI-II and the TGDS are equally reliable when administered to patients with PD. Our finding of sound test-retest reliabilities of the BDI-II and the TGDS are consistent with those of previous studies when these two measures are applied to other populations [20, 21, 29].

Our results showed that the MDCs of the BDI-II and the TGDS were 8.7 points and 5.4 points, respectively. The results suggest that a score of ≥ 9 for the BDI-II and one of ≥ 6 points for the TGDS are needed to announce a real change (beyond random measurement error) between repeated measurements. Our results can be viewed as a threshold reference for the BDI-II and the TGDS to help clinicians and researchers reasonably and confidently determine real change between repeated measurements for patients with PD.

We further compared the proportion of random measurement error relative to possible maximal score (i.e., MDC%) of these two measures. The MDC% of the BDI-II (13.8%) and that of the TGDS (18.0%) were acceptable to nearly excellent [24], suggesting that both measures are suitable for detecting a true change between two successive tests for a single patient with PD in a clinical setting. Based on the number of items and the MDC%, the BDI-II seems to be more appropriate for use in detecting the status of depression of patients with PD in clinical settings.

In comparing our results to those of a previous study [20], we found that both studies showed that the test-retest reliability of the BDI-II for patients with PD was high, with ICCs above 0.86, further supporting the reliability of the BDI-II in patients with PD. On the other hand, the MDC in our research (8.7 points) was somewhat greater than that of a previous study [20] (3.3 points). However, the characteristics of the sample (e.g., the number and severity of disease of participants) and detailed data (e.g., the mean scores and standard deviation of the test and the retest) included in the data analysis of test-retest reliability and MDC were not clearly reported in that study [20]. These shortcomings limit the generalization of those results, hindering any further comparison between our results and those of the previous study [20]. However, our finding of an MDC of 8.7 points could be viewed as a high standard to detect a real change between two sessions in patients with PD.

In terms of heteroscedasticity, the Bland-Altman plot (Fig 1) shows an obvious systematic trend for the BDI-II, and the Pearson’s r values between the absolute value of difference and the mean of the BDI-II were > 0.3, indicating the data were heteroscedastic (i.e., the mean and the difference of each pair of repeated measurements increased or decreased simultaneously). According to Flansbjer et al., if the data are heteroscedastic, a fixed value of MDC is not a proper threshold for determining a real change between repeated measurements because the amount of random measurement error depends on the initial test score of the measure [13]. For example, if a patient has an initial BDI-II score of 30 points, a change of more than 4.1 points (30×0.138) is needed to indicate a true change. The MDC of 8.7 points could be considered as the highest criterion for detecting a real change between successive tests of the BDI-II. As for TGDS, Pearson's r was 0.3, just close to the criterion of the presence of the heteroscedasticity. If prospective users concern such an issue, the users can use the same rules applied to the BDI-II mentioned above to estimate the true change between successive tests of the TGDS. These results should be helpful for clinicians to interpret the changes between repeated measurements for single patients more accurately.

According to the results of paired t tests between the test and retest sessions of the BDI-II and the TGDS (p > 0.05), no systematic bias was found in this research.

Regarding the issue of the patients lost to follow-up in this study, the number of patients lost to follow-up was five. Their mean score (SD) of the BDI-II was 2.6 (0.9), which was significantly different from that of the other 70 participants. We could not accurately estimate the degree to which the loss of the 5 patients to follow-up influenced our results. However, the proportion of patients lost to follow-up (5) to all participants (75) was about 6.7%, and the variation of these patients seemed small (SD = 0.9), implying a limited effect of the loss of the 5 patients to follow-up on the robustness of our results.

In the current study, we focused on determining the MDC to depict the extent of random error and a threshold of statistical significance for the BDI-II and the TGDS. However, in a clinical context, the minimal important difference (MID), which is the degree of a change meaningful to a patient, is equally crucial in decision making in treatment planning [30]. To improve the utility and applicability of the BDI-II and the TGDS for patients with PD, future research to investigate the MID of the two measures is warranted.

This study has one limitation. The characteristics of our sample (a convenience sample and participants with mild to moderate severity) might threaten the generalizability of our findings. Future research could enroll more patients with a more even distribution (e.g., from mild to severe disability) to cross-validate our findings.

## Conclusion

Both the BDI-II and the TGDS are reliable measures of the severity of depression in people with PD. In addition, both measures have acceptable to nearly excellent random measurement error. These results imply that the BDI-II and the TGDS are suitable for use in research and clinical settings to detect real change in a single patient with PD.

## Supporting information

S1 Dataset Dataset of the study. (XLSX)
